# First in-flight synchrotron X-ray absorption and photoemission study of carbon soot nanoparticles

**DOI:** 10.1038/srep36495

**Published:** 2016-11-24

**Authors:** F.-X. Ouf, P. Parent, C. Laffon, I. Marhaba, D. Ferry, B. Marcillaud, E. Antonsson, S. Benkoula, X.-J. Liu, C. Nicolas, E. Robert, M. Patanen, F.-A. Barreda, O. Sublemontier, A. Coppalle, J. Yon, F. Miserque, T. Mostefaoui, T. Z. Regier, J.-B. A. Mitchell, C. Miron

**Affiliations:** 1 Institut de Radioprotection et de Sûreté Nucléaire (IRSN), PSN-RES, SCA, LPMA, Gif-Sur-Yvette, 91192, France; 2 Aix Marseille Univ, CNRS, CINAM, Marseille, France; 3 Synchrotron SOLEIL, Saint Aubin, BP 48, Gif-sur-Yvette Cedex, 91192, France; 4 Physical Chemistry, Freie Universität Berlin Takustr. 3, D-14195 Berlin, Germany; 5 School of Physics and Nuclear Energy Engineering, Beihang University, Beijing 100191,China; 6 Nano and Molecular Systems Research Unit, Molecular Materials Research Community, Faculty of Science, P.O. Box 3000, 90014 University of Oulu, Finland; 7 NIMBE/CEA/CNRS/Université Paris-Saclay/Laboratoire Edifices Nanométriques, CEA Saclay, Gif-sur-Yvette Cedex, 91191, France; 8 Normandie Univ, INSA Rouen, UNIROUEN, CNRS, CORIA, 76000 Rouen, France; 9 CEA/DEN/DPC/SCCME/Laboratoire d’Etude de la Corrosion Aqueuse, CEA Saclay, Gif-sur-Yvette Cedex, 91191, France; 10 Laboratoire de Physico-Chimie des Matériaux et Catalyse, Faculté des Sciences Exactes, Université de Bejaia, 06000 Bejaia, Algérie; 11 Canadian Light Source, Saskatoon, SK S7N 2V3, Canada; 12 Institut de Physique de Rennes, UMR 6251, Université Rennes 1, Rennes Cedex, 35042, France; 13 Extreme Light Infrastructure - Nuclear Physiscs (ELI-NP), “Horia Hulubei” National Institute for Physics and Nuclear Engineering, 30 Reactorului Street, RO-077125 Măgurele, Jud. Ilfov, Romania

## Abstract

Many studies have been conducted on the environmental impacts of combustion generated aerosols. Due to their complex composition and morphology, their chemical reactivity is not well understood and new developments of analysis methods are needed. We report the first demonstration of in-flight X-ray based characterizations of freshly emitted soot particles, which is of paramount importance for understanding the role of one of the main anthropogenic particulate contributors to global climate change. Soot particles, produced by a burner for several air-to-fuel ratios, were injected through an aerodynamic lens, focusing them to a region where they interacted with synchrotron radiation. X-ray photoelectron spectroscopy and carbon K-edge near-edge X-ray absorption spectroscopy were performed and compared to those obtained for supported samples. A good agreement is found between these samples, although slight oxidation is observed for supported samples. Our experiments demonstrate that NEXAFS characterization of supported samples provides relevant information on soot composition, with limited effects of contamination or ageing under ambient storage conditions. The highly surface sensitive XPS experiments of airborne soot indicate that the oxidation is different at the surface as compared to the bulk probed by NEXAFS. We also report changes in soot’s work function obtained at different combustion conditions.

The impact of combustion aerosols on the earth’s climate is a still poorly understood subject. All recent reports agree that they have a significant radiative contribution to global warming, yet the quantitative estimations contain large uncertainties^1^. The interactions of soot aerosols with the environment and most importantly their reactions with atmospheric water are assumed to be controlled by their surface reactivity (oxidation, adsorption of water molecules), which might depend on the initial fuel composition and the combustion conditions. Considerable efforts have been conducted to study the evolution of size, morphology and chemical composition of aerosol soot particles during atmospheric ageing^2^,^3^,^4^. Similar investigations have been carried out in order to describe the evolution of soot microstructure^5^,^6^, morphology or size distribution^7^,^8^ during high temperature oxidation. The characterization of soot is also essential for predicting the behaviour of filtration systems under fire conditions^9^ that can occur in industry and potentially in nuclear facilities. Most of these studies have been achieved on freshly emitted airborne soot particles in the aerosol phase in order to be the most representative of the real *in-situ* conditions, and this intense effort for developing in-flight analysis methods has allowed a better description to be obtained of the evolution of the physical and chemical properties of soot particles under complex conditions. However, an in-depth knowledge of oxidation and gas/particle interactions calls for a nanoscale description of the surface of the soot particles. Achieving surface sensitivity is one of the main features of X-ray Photoelectron Spectroscopy (XPS), a technique widely used for studying the chemical composition of solid materials. XPS has been applied over the last two decades on soot particles^10^,^11^, along with Near Edge X-ray Absorption Fine Structure (NEXAFS)^12^,^13^,^14^, a surface-sensitive technique that uses the photoelectric effect for detection purposes. These methods have also been successfully used in the field of water/soot interactions^15^,^16^, atmospheric ageing^17^,^18^,^19^ or high temperature oxidation^20^,^21^. However, XPS and NEXAFS experiments are generally performed on solids or thin films and, until now, concerning soot characterization, they were restricted to soot films deposited on substrates. In this case, contamination of the samples in the ambient air may have occurred between the moment when they are produced and the moment when they are analysed, raising the question of their ageing under atmospheric conditions^17^,^22^, and hence the relevance of such methods for studying soot reactivity when working with soot samples deposited on a substrate.

The development of experiments allowing freshly produced nanoparticles to be characterized in the airborne phase is highly desirable. It not only avoids ambient contamination of the aerosol, but it can also allow transient, metastable species to be detected during the particle growth, and highly reactive species present on the soot surface to be characterized. Ravagnan *et al.*^23^ have reported a time-resolved NEXAFS analysis of free carbon aggregates produced from graphite sputtered by a pulsed He-plasma plume under Ultra-High Vacuum (UHV) conditions. An unstable carbon *sp* component was detected in the airborne phase, which rapidly converted to *sp*^2^ hybridized carbon, whereas this *sp* signature was hardly observable on a deposited film. Recently, the development of aerodynamic lens (ADLS) technology has allowed focused beams of particles to be produced, which, coupled with a X-ray free electron laser, has provided information on the morphology and composition of single soot particles^24^,^25^. In addition, using an ADLS device, similar to that designed by Jayne *et al.*^26^ based on the original design of Liu *et al.*^27^,^28^, XPS experiments on non-supported aerodynamically focused SiO_2_ model nanoparticles have been performed at different synchrotron facilities^29^,^30^. More recently, Sublemontier *et al.*^31^ and Benkoula *et al.*^32^ have investigated the oxidation state of silicon nanoparticles and the hydration properties of TiO_2_ nanoparticles, respectively. They both have used an ADLS device recently designed^33^ and characterized^34^ at the PLEIADES beamline of SOLEIL, the French synchrotron radiation facility. In the present work, we have adapted this device to study carbon soot particles, in order to demonstrate the feasibility of in-flight NEXAFS and XPS analysis of this kind of aerosol. The experiments presented here are not only *in-situ*, they are also *in-flight* measurements. Combining *in-situ* and *in-flight* analysis allows freshly emitted soot (*in-situ*) to be studied in the airborne phase (*in-flight*). Another goal of this work is to compare the spectra obtained in the aerosol phase with those obtained on the same soot supported on a substrate. We will show that ageing after a moderate exposure at ambient air, only slightly modifies the soot surface – inducing a low level of surface oxidation - leaving most of the carbonaceous surface and the inner nanostructure of the primary particles intact. This demonstrates that the analysis methodology involving supported samples is also relevant for soot particles having a limited reactivity in ambient air.

## Methods

**Figure 1:**
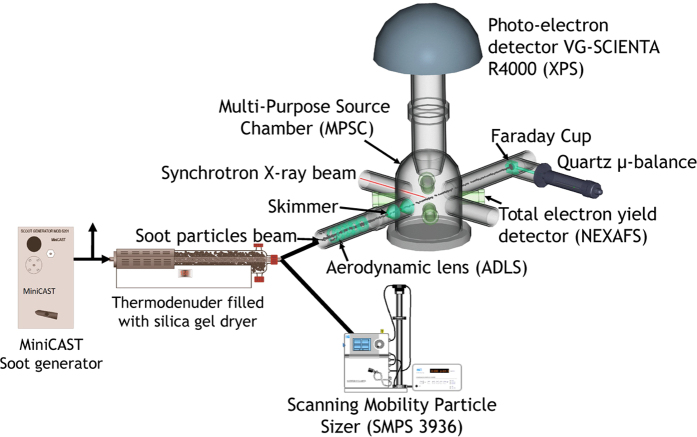
Schematic view of the experimental setup used in our synchrotron radiation-based XPS and NEXAFS experiment of non-supported soot particles at the PLEIADES beamline.

The experiment is schematized in Fig. 1. The soot particles were produced by a propane diffusion burner (miniCAST Jing Ltd.) and transported through a tube heated to 200 °C to prevent any condensation on the walls. Soot generated by the miniCAST has been widely investigated in terms of size distribution, morphology and chemical composition^35^,^36^,^37^,^38^,^39^, but to the best of our knowledge, no analysis of the surface composition has yet been carried out. The miniCAST is known to satisfactorily mimic the physical and chemical behaviour of many kinds of soot particles, such as those emitted by diesel^40^ or aircraft engines^41^,^42^, and the small size of the miniCAST makes it practical to implement on a synchrotron beamline. By changing the fuel-to air ratio of the flame, it is possible to produce soot particles of different size, morphology and organic-to-total carbon ratio (OC/TC). Three “set points” of the burner were studied, named hereafter CAST1, CAST2 and CAST3, corresponding to the operating conditions reported in Table 1. The pre-dilution nitrogen, the quench nitrogen, and the dilution air flow rates were kept constant at 0 L/min, 7.5 L/min and 20 L/min, respectively. Downstream of the miniCAST, a flow rate of 0.6 L/min was injected through a thermodenuder (TSI 3065, temperature up to 400 °C) filled with silica gel to avoid water condensation and to remove water potentially physisorbed on the soot surface. The flow was then divided in two parts, the first being used for real-time size distribution measurements with a Scanning Mobility Particle Sizer (SMPS) (TSI 3936L), while the second part was injected into the XPS/NEXAFS analysis chamber. Microstructure and morphology of the soot particles were also characterized off-line using High Resolution Transmission Electronic Microscopy (HRTEM) and TEM^22^, on samples collected by filtration onto carbon-coated copper grids (AGAR Scientific). Organic carbon-to-total carbon ratio OC/TC was also determined using a thermo-optical analyser from Sunset Lab^43^. according to the IMPROVE_A protocol^44^, on particles sampled on quartz fibre filters (Pall Tissuquartz 2500 QAT-UP, 47 mm in diameter). The filters were baked at 850 °C for 1 hour to desorb any organic content potentially absorbed on these filters during their storage under atmospheric conditions.

The experiments were carried out using the Multi-Purpose Source Chamber (MPSC, hereafter the “analysis chamber”)^33^ of the ultra-high resolution^45^ soft X-ray beamline PLEIADES (10–1000 eV) at the SOLEIL synchrotron radiation facility (Saint-Aubin, France). The carbon 1s XPS spectra were recorded using a 30° aperture wide-angle lens VG-Scienta R4000 electron energy analyser, at an incident photon energy of 350 eV, and recording photoelectrons in the kinetic energy range from 45 to 75 eV. In this range, the electron inelastic mean-free path (IMFP) is estimated to be 0.5 nm^46^, and XPS recorded under these conditions probes the very surface of the soot particles. For each set point, the NEXAFS spectra at the carbon K-edge were recorded directly after XPS in the total electron yield (TEY) mode, using a positively polarized micro-channel plate detector (home-made detector with a 50 ohms adapted anode). The depth probed with the TEY method is about 5.0 nm^47^, i.e. larger than for XPS. Since the mean radius of the soot primary particles is around 15 nm (Table 1), the NEXAFS signal probes their bulk. The particles produced by the miniCAST were focused into the analysis chamber through the ADLS, equipped with a skimmer specifically designed to avoid its clogging (Fig. 1). The stability of the particle concentration in the analysis chamber was monitored with a Faraday cup connected to an electrometer, the cup being placed on the axis of the focused nanoparticle beam. We performed a pre-alignment of the particle beam with the X-ray beam by injecting an atomized, colloidal suspension of SiO_2_ nanoparticles into the aerodynamic lens. Since the properties of the nanoparticle beam are strongly dependent on the nature, size and shape of particles, the alignment was further optimized when the soot was injected. The incident X-ray energy calibration was achieved using the C1s → π* transition of CO_2_ at 290.77 eV +/− 0.02 eV measured by Antonsson *et al.*^48^ on the same beamline. The kinetic energy recorded by the electron analyser was calibrated with respect to the vacuum level using the C 1s binding energy at 297.63 ± 0.01 eV of gaseous CO_2_^49^ resulting from the propane combustion and emitted into the stream of the nanoparticle beam. The resolution of the C1s XPS measurements was estimated to be 250 meV, including X-ray bandwidth and electron spectrometer resolution.

NEXAFS and XPS measurements were also performed on substrate supported soot (on bare silicon windows from UQG optics) deposited using exactly the same set points, thus allowing a direct comparison with the results taken on the airborne soot. These experiments were carried out at the high-resolution Spherical Grating Monochromator (SGM) beamline (11 ID-1) of the Canadian Light Source (CLS) synchrotron radiation facility, Saskatchewan, Canada. The samples were prepared two weeks before the synchrotron experiments and kept under nitrogen atmosphere in sealed bags containing silica gel packets, and opened just before their transfer into the UHV analysis chamber. During the transfer, the samples were exposed to ambient air for several minutes. As in the NEXAFS experiments at SOLEIL, the data at the CLS were recorded in the TEY mode using the drain photocurrent. The spectra were normalized to the incident beam intensity using the C1s TEY spectrum of a clean Au foil. A highly ordered pyrolytic graphite (HOPG) sample was used as reference for calibration and interpretation of soot spectra, and was cleaved in air immediately prior to its introduction into the UHV chamber. To allow the SOLEIL and the CLS NEXAFS data to be directly compared, the pre-edge was set to zero and the data were normalized by their integral value. Complementary laboratory XPS experiments were performed at the CINaM, on another batch of the same soot produced at the same set points, deposited on gold-plated silicon substrates (UQG optics). The samples were also prepared two weeks before the XPS experiments, and stored and transferred with the same procedure as for the NEXAFS experiments at the CLS. We collected the laboratory XPS data using Mg Kα radiation (1253.6 eV) delivered by an X-ray source (PSP Vacuum Technology) operated at 200 W without monochromatization. The photoelectron spectra were recorded using an electrostatic hemispherical analyser (Resolve 120) with 5-channel detection (PSP Vacuum Technology), with a pass energy of 50 eV for the survey spectra and 20 eV for the detail spectra. The purpose of these complementary experiments was to quantify the oxygen concentration for the supported soot, rather than to perform a detailed spectroscopic analysis. Due to more energetic photon excitation and detection angle, the inelastic mean-free path in these laboratory XPS experiments was 1.3 nm, higher than for the in-flight XPS data recorded at SOLEIL (0.5 nm), and closer to the 5.0 nm probed with NEXAFS in the TEY mode (SOLEIL and CLS).

## Results and Discussion

**Figure 2:**
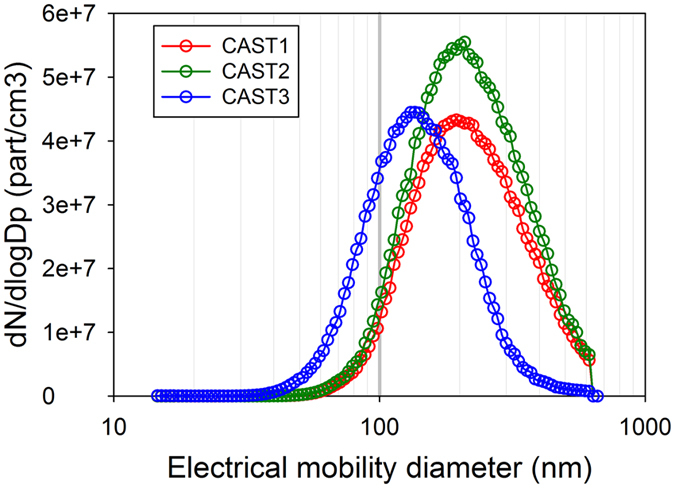
Size distribution of the soot samples injected in the analysis chamber in the three selected experimental conditions (set points), as measured with the SMPS.

**Figure 3:**
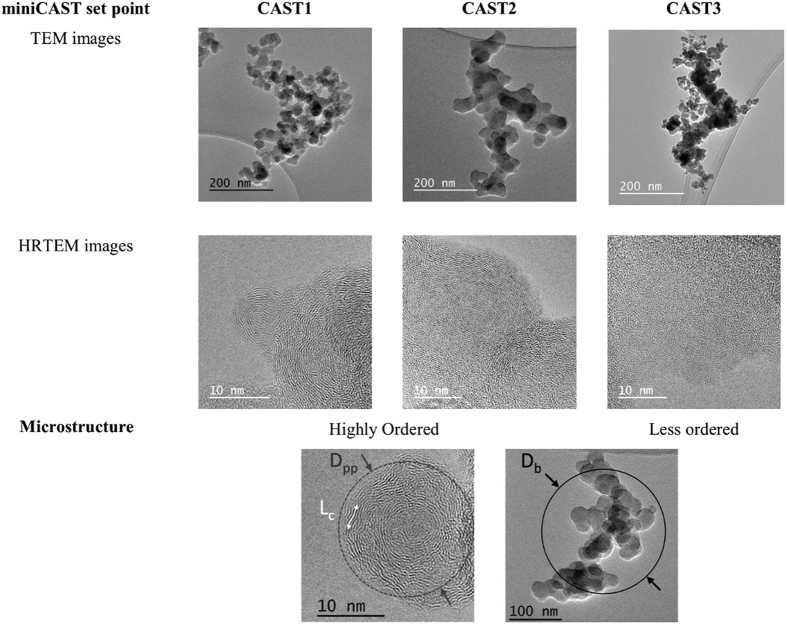
TEM and HRTEM images of soot particles produced by the miniCAST for each set point and analysed off-line on samples deposited on carbon-coated copper grids. The crystallites length (L_c_), the primary particle diameter (D_pp_) and the electrical mobility diameter (D_b_) of a soot aggregate are shown at the bottom of the figure.

It is well known that soot particles are fractal aggregates composed of ultrafine carbonaceous primary particles. Considering this large range of characteristic size, these particles could be described by several characteristic lengths or diameters. Considering the microstructure scale, the crystallites composing the bulk material of soot are generally described by their length L_c_. At the upper scale, primary particles are generally spherical, partially inter-penetrated and are described according to their geometrical diameter D_pp_ measured in most cases on TEM images. At the largest size scale and due to their complex morphology, soot aggregates are usually characterized according to their equivalent electrical mobility diameter D_b_ (diameter of a spherical particles carrying one elemental charge and demonstrating the same electrical mobility as the corresponding aggregate). Figure 2 shows the electrical mobility diameter (D_b_) provided by the SMPS. This is a measure of the size distribution of the soot aggregates. It is significantly smaller for CAST3 (138 nm) than for CAST1 and CAST2 (211 nm). Typical TEM micrographs of the aggregates and their corresponding microstructure at higher magnification (HRTEM) are shown in Fig. 3. Figure 3 also presents a sketch of a soot aggregate with its corresponding characteristics length or diameter. The aggregate morphology is characteristically fractal, and the primary particles forming the aggregates show a more ordered (CAST1, CAST2) or a less ordered (CAST3) microstructure. Determined by TEM during off-line measurement on samples on carbon-coated copper grids, the diameter of the primary particles (D_pp_) is similar for CAST1 and CAST3–27 and 30 nm, respectively-, and slightly larger for CAST2 (36 nm) (Table 1). The organic-to-total carbon OC/TC ratios are also indicated in this table, and range from 4% to 87%.

### NEXAFS

**Figure 4:**
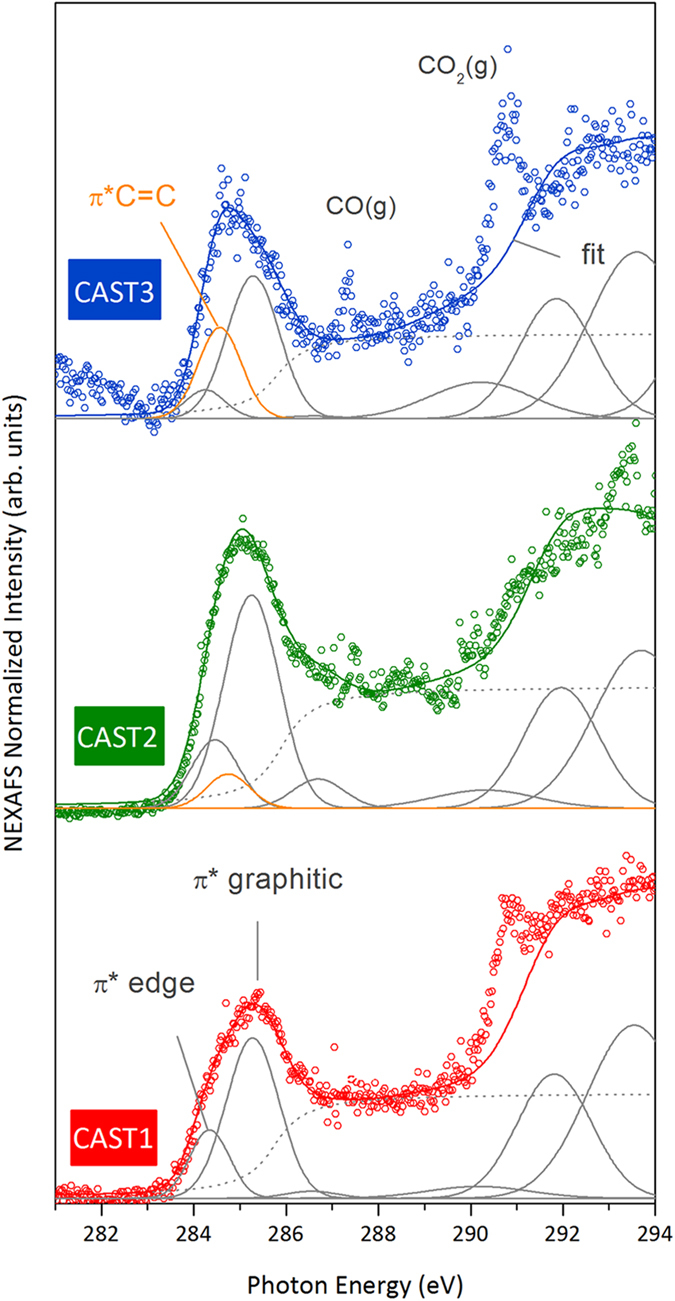
C1s NEXAFS spectra recorded on the aerosol phase at the PLEIADES beamline at SOLEIL, for different set points of the miniCAST aerosol source. The data have been vertically shifted for display.

Figure 4 shows the in-flight NEXAFS spectra for the three set points. These spectra are typical of nanostructured graphitic soot^14^. They show a main peak at 285.3 eV, labelled “π*graphitic”, corresponding to the C1s → π* excitation of the *sp*^2^-hybridized carbon in the graphitic planes of the primary particles. These so-called “crystallite” planes are observed as dark lines, or “fringes”, on the HRTEM images of highly ordered soot (CAST1 and CAST2) (Fig. 3). They are organized to a greater or lesser degree, into so-called “turbostratic”, spherical, concentrically onion-like structures. At the low energy side of the π*graphitic, one can distinguish a shoulder at 284.5 eV, well visible on the CAST1 spectrum. It is labeled “π*edge”, and results from the C1s → π* excitation related to the *sp*^2^-hybridized carbon atoms located at the edges of the crystallites^50^,^51^,^52^,^53^,^54^,^55^,^56^. These two transitions are fitted with Gaussian functions. An additional Gaussian contribution at 284.7 eV is necessary to fit the spectra of samples CAST2 and CAST3 but is not needed in the CAST1 sample. It corresponds to the C1s → π* excitation of -C = C- functionalities of unsaturated organic molecules^57^ and is labeled π* C = C. Its intensity is about 2 times higher in the CAST3 than in the CAST2 samples. It is related to the “organic phase” contained in the soot, and follows the increase in the OC/TC ratio from CAST1 to CAST3 (Table 1). FTIR analysis performed on miniCAST samples produced at the same set points^58^ revealed a band associated with the -C = C- stretching vibrations that also increase in intensity with the organic content.

In nanostructured graphitic compounds, the intensity ratio R = π*(edge)/π*graphitic corresponds to the ratio of carbon atoms located at the periphery to those located inside a crystallite^51^,^54^. R decreases as the crystallite size increases, and falls to zero both for graphite and for an infinite graphene layer^51^,^54^. From R, we can deduce the crystallite sizes by numbering the edge-type and core-type carbon atoms on a two-dimensional graphene model^59^. For CAST1 R = 0.32, which corresponds to a crystallite size of 3.3 nm, in good agreement with the TEM values (2.8 nm, Table 1). For CAST2 and CAST3, if we calculate R in the same way, we obtain crystallite sizes far larger than the HRTEM values (2.7 nm for CAST2 and 0.6 nm for CAST3 - Table 1). However, when we include the organic phase by calculating the intensity ratio [π*edge + π*C = C]/[π*graphitic], we obtain respective sizes of 3.0 and 0.7 nm for CAST2 and CAST3, in excellent agreement with the HRTEM values. In this case, a -C = C- moiety replaces an edge atom, and must be counted as such. This is a clue that these moieties are connected to the edge atoms. Furthermore, if these organics had included more than one -C = C- functionality, this calculation of R would have overestimated the number of edge atoms to which the -C = C- are attached, and the crystalline sizes would appear smaller. Thus, the organic phase, attached to the edge of the crystallites, is made up of aliphatic chains with a single -C = C- on average.

In this spectral range, a small additional contribution at 286.6 eV is also necessary to properly fit the right part of the π* graphitic transition for the CAST2 sample, but is not needed for the CAST1 and CAST3 samples. This transition is related to the C1s → π* of C-OH phenol groups^16^,^60^.

The continuum step, starting at around 287 eV and giving rise to a flat spectral region up to 290 eV, is specific to graphite and graphite-like materials^61^,^62^,^63^. It is simulated by an arctangent function (dotted lines). In this range, a sharp peak is also observed at 287.3 eV on the CAST3 spectrum; it corresponds to the C1s → π* excitation of the CO molecule in the gas phase^64^. CO is emitted by the combustion of propane and flows in the stream of the soot beam, and is more predominant for set points with low oxidation air flow rate (CAST2 and CAST3). We assume that the few points out of the spectra observed in the CAST1 and CAST2 data also come from CO. Likewise, the strong peak at 290.8 eV observed on the CAST1 and CAST3 spectra result from the C1s → π* excitation of the CO_2_ molecule in the gas phase^64^, also emitted by the burner. This transition overlaps a second threshold at around 290 eV, very steep in graphite^61^ but strongly blurred in graphite-like materials containing defects^63^. In previous work^14^, we have simulated this second threshold using a broadened σ* exciton contribution at 291.8 eV, followed by a σ*graphitic resonance at 293.6 eV associated with carbon bonds in the hexagons of the graphene layers. The blurred character of the threshold was also well fitted by a broad Gaussian function at 290.2 eV, assigned to structural defects within the graphene plane such as hexagon-pentagon defects^65^, giving some density of state at this energy^66^,^67^. This also provides a satisfactory approximation to the spectra of the aerosol soot presented here, as shown by the results of the fits plotted in Fig. 4 for the three set points (full lines; the CO_2_ contribution is not count).

**Figure 5:**
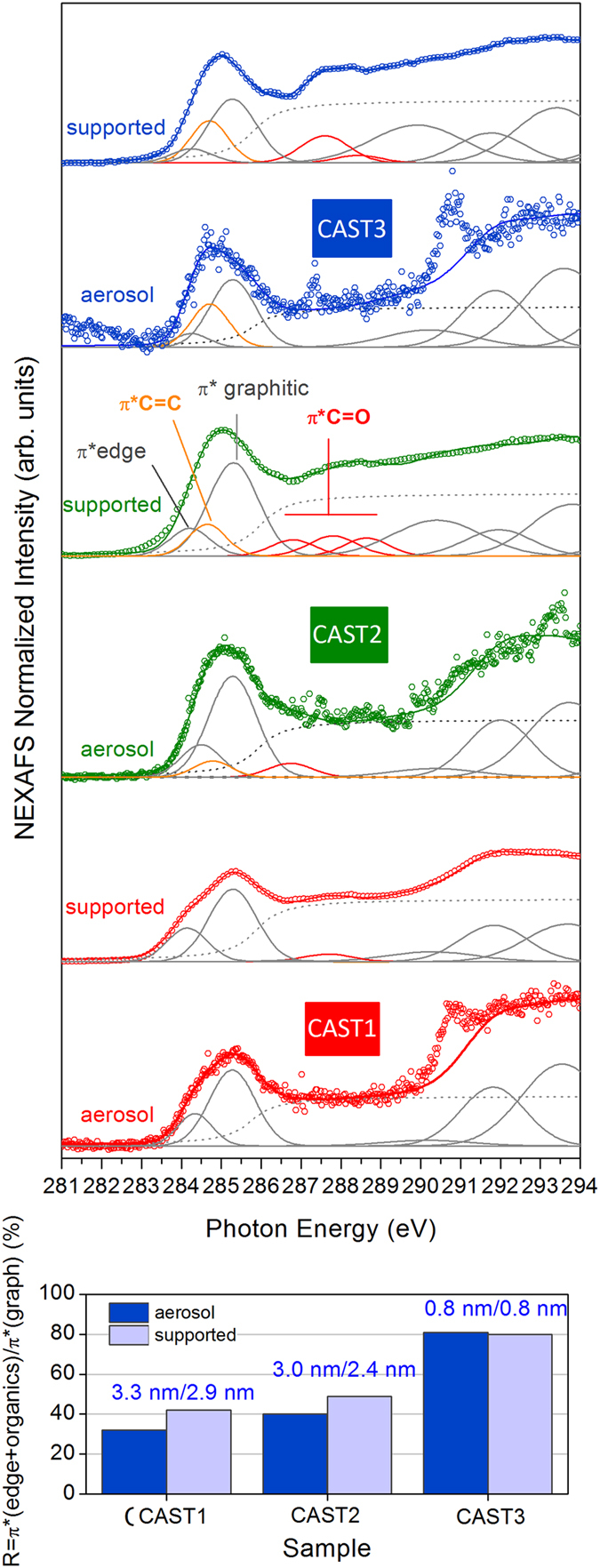
Top: Comparison between the C1s NEXAFS spectra recorded on the aerosol phase on the PLEIADES beamline at SOLEIL with those recorded on the supported samples on the SGM beamline at CLS. Bottom: R values for the three set points and the two phases; the inferred crystallite sized are also indicated (see text).

Figure 5 (top) compares the C1s NEXAFS spectra of the aerosol recorded in-flight at SOLEIL with those recorded on the supported soot at the CLS. The spectra of the supported samples have the same overall shape than the corresponding aerosols. The data obtained on the deposited soot are obviously free from gas phase CO and CO_2_. They also have less noise, the density of material being higher than in the beam of nanoparticles. The two phases show very similar C1s → π* edge, graphitic and organic transitions. Figure 5 (bottom) presents the intensity ratios R, plotted as columns, and indicates the crystallite sizes calculated from R. The ratio, hence the sizes, are very close for the two phases. One strong difference between the supported and the aerosol phases is observed for the CAST3. The spectrum of the supported CAST3 sample clearly shows strong carbon oxide transitions at 287.8 eV, absent in the aerosol phase. These transitions are fitted with two C1s → π* C = O excitations at 287.6 eV (aliphatic/aromatic carbonyl -C = O) and 288.4 eV (acids/carboxyl –COOH). Less marked oxide contributions are observed for the supported CAST1 and CAST2. Although this energy domain is quite noisy in the aerosol phase, and furthermore contaminated by CO (gas), these oxidations are absent in the spectra of the CAST1 and CAST2 aerosol phase. This is a strong hint that oxidation of the supported soot occurs by exposure to the ambient air. Our complementary laboratory XPS experiments (not presented) show that the oxygen contamination at the surface of the supported samples is 4 at.% (CAST1), 4 at.% (CAST2) and 10 at.% (CAST3). The CAST3 sample is more contaminated than the CAST1 and CAST2 samples, in agreement with what qualitatively inferred from the NEXAFS data of the supported soot.

### XPS

**Figure 6:**
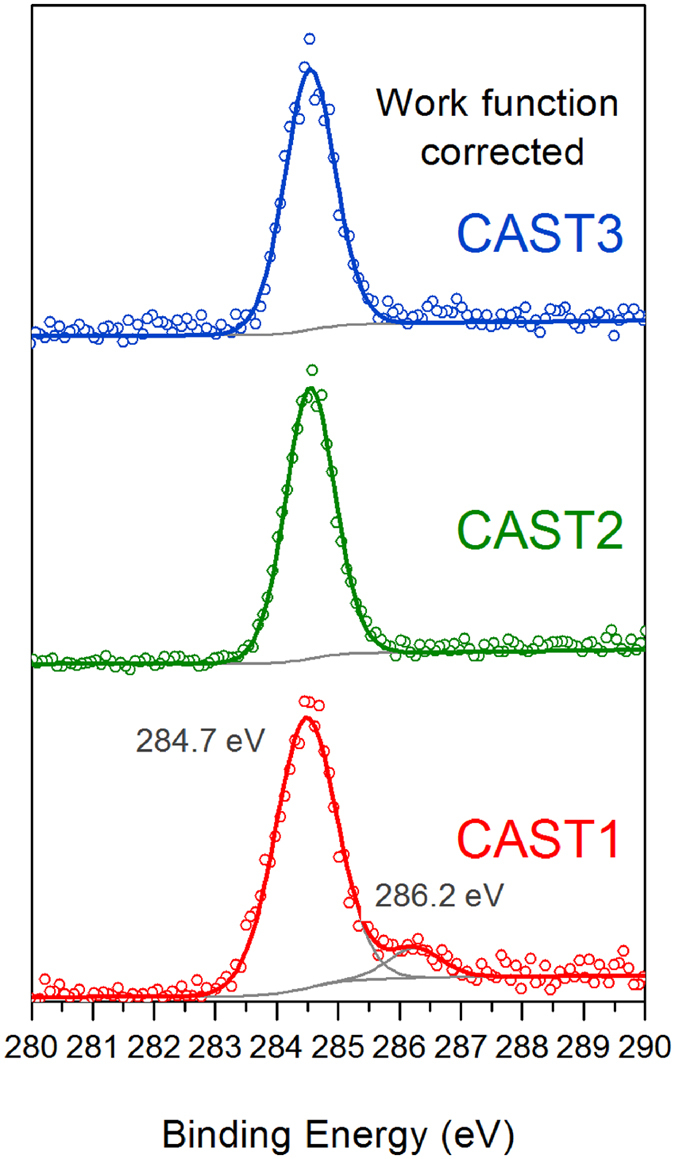
Comparison of C1s XPS spectra recorded on the aerosol phase at the PLEIADES beamline at SOLEIL for each set point.

The XPS spectra obtained for the airborne particles are shown in Fig. 6. Data are also displayed in the supporting information (Figure S1) using a binding energy scale calibrated against the C1s line of CO_2_ in the gas phase (297.6 eV), and thus we obtain the binding energies with respect to the vacuum level. The line at around 284–285 eV (289 eV in Figure S1) is assigned to the C1s emission of soot. It disappears if a particle filter is set downstream to stop the particles in the beam before the analysis chamber (not shown). Since the HRTEM and NEXAFS results indicate that the particles are graphitic, we would expect the C1s line to lie around 284.7 eV, as typical of *sp*^2^-hybridized carbon^14^. The CO_2_ gas calibration allows us to access the work function (WF) of the soot particles directly, namely, the binding energy shift ~289 eV to 284.7 eV is due to the WF of the soot, which is to be seen as the energy necessary for the photoelectron to escape the particle.

Figure 6 presents the XPS data corrected for the respective WF of the soot of different set points (i.e., the energy of the main C1s line is now set at 284.7 eV). Figure S1 in the Supplementary Material shows the same spectra referenced to the vacuum level. The spectra have been fitted considering a main peak at 284.7 eV corresponding to *sp*^2^-hybridized carbon in graphitic planes (and in the organic phase, when present), and a second peak around 286.2 eV, characteristic of oxidized carbon atoms with a single C-O bond, as in hydroxyl, phenol or alcohol (C-OH), or in the ether group (C-O-C)^68^,^69^. The CAST2 and CAST3 spectra have a single contribution at 284.7 eV and no contribution at 286.2 eV, showing the absence of oxidation at the surface for the airborne particles produced at these two set points. If no surface oxidation is detected on CAST2 with XPS, it should be remembered, however, that few C-OH groups were detected with in-flight NEXAFS, which probes the bulk. They might also be present at the surface, but are not sufficiently concentrated to be detected by XPS. Concerning CAST1, an oxide contribution (9 at.%) is observed at 286.2 eV. This was not seen with in-flight NEXAFS, indicating that these species remain at the very surface and are not present in the bulk. Note that the CAST1 soot has the lowest OC/TC ratio (4%), showing that the surface oxidation is not related to the organic content.

The XPS data reported for “reference sample” showed that the oxygen content was 10 at.% for CAST3, and 4 at.% for CAST1 and CAST2. OC/TC measurements indicate that, however, the CAST2 sample has an organic content 10 times larger than for CAST1, showing that the soot oxidation is not related to the organic phase content. NEXAFS provides some information on the composition of this organic phase, which is mostly made up of organic chains with a single -C = C- moiety, which should not be very reactive to the ambient oxidizing species (water, oxygen) to which they have been exposed. The peculiarities of the CAST3 sample are its very small carbonaceous crystallite size, its lower structural order, and its high organic content compared to the CAST1 and CAST2 samples. Furthermore, the NEXAFS data (Fig. 5) show that the intensity of the π*(edge) of the CAST3 sample is lower than for CAST1 and CAST2, while that of the π*(organic) is higher: in CAST3, the saturation of the edge-type carbon atoms by organic moieties reduces the amount of edge-type carbon atoms. One might suppose that the sensitivity to oxidation is related to the edge concentration, because soot usually contains free carbon radical at these edge sites^70^ that can easily react with water and oxygen. However, this is contradicted by the higher oxidation rate of the supported CAST3. Therefore, such higher oxidation may be rather due to a higher accessibility of the oxidizing molecules to these edge sites, most likely related to the poor structural order of the sample.

Finally, we would like to compare the WF values found on the airborne soot with those obtained for carbon particles reported in literature (Table 2). For graphite, WF ranges from 4.35 eV to 4.63 eV. Li *et al.* have reported similar values for various soot particles, ranging from 4.34 to 4.74 eV^71^. WF is a property that strongly depends on the surface of the material. For nanoparticles, surface contamination (oxidation, organics) and morphology at the nanoscale are key parameters. Fabish *et al.*^72^ have shown that the work function of soot particles increases with their oxygen content. This is consistent with our data, the oxygen contamination measured by XPS in the aerosol phase being undetectable for CAST2 and CAST3 samples, and being of 9 at.% for the CAST1 sample, for which the work function is indeed the largest (4.77 eV). Concerning morphology, the WF of aggregates of silver nanoparticles have been investigated by Zhou *et al.*^73^,^74^. They show that it is the size of the primary particles that controls the WF, and not the size of the whole aggregate. Likewise, the WFs determined in the present work are also not correlated with the size of the soot aggregates D_b_ (Fig. 2 and Table 1).

**Figure 7:**
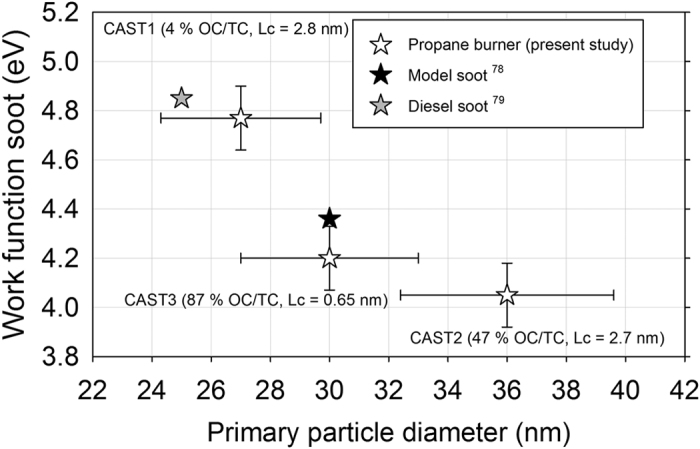
Work function of soot as a function of the primary particle diameter.

The evolution of the WF as a function of primary particle diameter of the soot (D_pp_) is shown in Fig. 7. They are quite close to those reported on Diesel soot by Matter *et al.*^78^ and Michelsen *et al.*^79^, and present a significant decrease with the primary particle diameters. This is the first time that such an evolution has been reported for soot particles. Wood^80^ demonstrated that, for metallic systems, the WF shifts observed from the bulk to nanoparticles can be explained by an electrostatic model based on image and Coulomb potentials of spherical geometry. This approach is only relevant for an infinitely conducting sphere without any surface contamination, which is not the case here. Along with size effects, the oxygen contamination may have enhanced the WF.

The work function of carbon particles is a very important parameter as it plays a role in maintaining arcing in degraded plastic insulation in electrical cables and in arcs between carbon electrodes^81^. The ability to be able to measure this parameter for free nanoparticles is therefore of great importance in the modelling of such phenomena. The WF of soot also has a significant influence in the interpretation of ionization produced by combustion processes in gasoline engines. It has been shown that ionization current data taken by post spark measurements using the spark plugs, can be used to monitor combustion conditions in these engines^82^,^83^. A theoretical study of this process^84^ attributed the ionization to the thermal ionization of nitric oxide which that has an ionization potential of 9.2 eV, and a very (unlikely) high concentration of this species was required to match the observed ionization current. What was ignored in this study was the presence of soot particles with a work function of between 4.05 and 4.77 eV (as demonstrated here) and since thermal ionization depends exponentially on the work function, this must have been the actual source of the ionization. Given this, and the influence of this parameter of soot particle size, it would seem worthwhile to revisit this subject with a view to a better understanding of the *in-situ* analysis of combustion conditions within gasoline powered engines.

## Conclusion

This study is the first demonstration of in-flight NEXAFS and XPS analysis on freshly emitted carbon soot in the aerosol phase. This analysis has been carried out on soot particles produced under various and controlled operating conditions of a miniCAST burner, a relevant source for mimicking diesel and aircraft exhaust and enabling the production of different chemical compositions in terms of organic contents (OC/TC ratio).

NEXAFS shows that this OC phase is made of aliphatic chains with one -C = C- moiety, which are attached to the edge of the graphitic crystallites. As emitted, the airborne particles are not oxidized in their bulk, although some surface oxidation is detected with XPS at one set point (CAST1). When the soot particles are deposited onto a substrate and stored in inert and dry conditions, they become slightly oxidized after exposure to ambient air even if exposure duration is reduced, but their inner structure is unchanged. Therefore, studying supported soot off-line is still relevant for describing the particles at the time when they are produced. The comparison of these supported and free aerosol data show that the latter method is clearly capable of preparing well-characterised soot particles beams. In-flight XPS analysis also allows the work function of the nanoparticles to be studied, and a significant influence of the primary particle diameter on the WF has been evidenced for the studied soot.

Improvement of the signal-to-noise ratio is still to be achieved, but these developments already open the way to future studies of model soot particles in the airborne phase, for studying their heterogeneous reactivity with oxidants (air, water) and hydrocarbons found in open burning processes, fire environments and at the exhausts of automotive and aircraft engines, and also for studying their water/ice nucleation properties, an important process in the atmospheric impact of these aerosols.

## Additional Information

**How to cite this article**: Ouf, F.-X. *et al.* First in-flight synchrotron X-ray absorption and photoemission study of carbon soot nanoparticles. *Sci. Rep.* **6**, 36495; doi: 10.1038/srep36495 (2016).

**Publisher’s note:** Springer Nature remains neutral with regard to jurisdictional claims in published maps and institutional affiliations.

## Supplementary Material

Supplementary Information

## Figures and Tables

**Table 1:** Operating conditions of the MiniCAST and characteristics of the emitted particles.

Setpoint	Propane (mL/min)	Air (mL/min)	**OC/TC (%)**	D_b_ (nm)	D_pp_ (nm)	Fractal dimension	Density (kg/m^3^)*	Crystallite length L_c_ (nm)
CAST1	60	1.5	**4**	211	27	1.73	1543	2.8
CAST2		1.15	**47**	212	36	1.75	1234	2.7
CAST3		1.0	**87**	138	30	1.79	1321	0.6

OC/TC: Organic-to-total carbon ratio, D_b_: count median electrical mobility diameter (SMPS) - D_pp_: count median primary particle diameter (TEM).

^*^From^39^.

**Table 2:** Work functions associated to different soot samples.

Source	Work function (eV)	Structure	OC/TC (%)	D_pp_ (nm)	Reference
Soot (CAST1)	4.77 ± 0.13**	Ordered	4	27	Present study
Soot (CAST2)	4.05 ± 0.13**	Ordered	47	36	
Soot (CAST3)	4.20 ± 0.13**	Less ordered	87	30	
Soot (butane)	4.34–4.40	?	?	?	71
Soot (benzene)	4.47–4.45	?	?	?	
Soot (naphthalene)	4.74–4.55	?	?	?	
Graphite	4.37–4.63	?	0	∞	
Assumption for soot	5.00	?	?	?	75
Graphite	4.60	Turbo	0	∞	76
Graphite	4.35	Turbo	0	∞	77
Diesel soot	4.85	?	?	25*	78
Model soot	4.37	Ordered	?	30	79

^*^From Müller *et al.*^21^.

^**^Considering errors due to the gas calibration (0.01 eV), the uncertainty in the value of 284.7 eV of the sp^2^-hybridized carbon in graphitic planes (0.1 eV), the fitting procedure used for determining the position of the C1s peak of each spectra (0.01 eV) and the step size of XPS spectra (0.08 eV).
