# Supplementary material for: First in-flight synchrotron X-ray absorption and photoemission study of carbon soot nanoparticles
Source: Sci Rep. 2016 Nov 24;6:36495. doi: 10.1038/srep36495 (PMC5121651; doi:10.1038/srep36495)
Supplement: Supplementary Information [file srep36495-s1.pdf]

First in-flight synchrotron X-ray absorption and photoemission study of carbon soot nanoparticles

F.-X. Ouf[1], P. Parent[2], C. Laffon[2], I. Marhaba[2], D. Ferry[2], B. Marcillaud[1], E. Antonsson[3, 4], S. Benkoula[3], X.-J. Liu[3], C. Nicolas[3], E. Robert[3], M. Patanen[3, 5], F.-A. Barreda[6], O. Sublemontier[6], A. Coppalle[7], J. Yon[7], F. Miserque[8], T. Mostefaoui[9], T.Z. Regier[10], J.-B. A. Mitchell[11], C. Miron[3,12]

[1]*Institut de Radioprotection et de Sûreté Nucléaire (IRSN), PSN-RES, SCA, LPMA, Gif-Sur-Yvette, 91192, France.*
[2]*Aix Marseille Univ, CNRS, CINAM, Marseille, France.*
[3]*Synchrotron SOLEIL, Saint Aubin, BP 48, Gif-sur-Yvette Cedex, 91192, France.*
[4]*Physical Chemistry, Freie Universität Berlin Takustr. 3, D-14195 Berlin, Germany.*
[5]*Nano and Molecular Systems Research Unit, Molecular Materials Research Community, Faculty of Science, P.O. Box 3000, 90014 University of Oulu, Finland.*
[6]*NIMBE/CEA/CNRS/Université Paris-Saclay/Laboratoire Edifices Nanométriques, CEA Saclay, Gif-sur-Yvette Cedex, 91191, France*
[7]*Normandie Univ, INSA Rouen, UNIROUEN, CNRS, CORIA, 76000 Rouen, France*
[8]*CEA/DEN/DPC/SCCME/Laboratoire d'Etude de la Corrosion Aqueuse, CEA Saclay, Gif-sur-Yvette Cedex, 91191, France.*
[9]*Laboratoire de Physico-Chimie des Matériaux et Catalyse, Faculté des Sciences Exactes, Université de Bejaia, 06000 Bejaia, Algérie.*
[10]*Canadian Light Source, Saskatoon, SK S7N 2V3, Canada.*
[11]*Institut de Physique de Rennes, UMR 6251, Université Rennes 1, Rennes Cedex, 35042, France.*
[12]*Extreme Light Infrastructure - Nuclear Physiscs (ELI-NP), "Horia Hulubei" National Institute for Physics and Nuclear Engineering, Reactorului Street, RO-077125 Măgurele, Jud. Ilfov, Romania.*

Correspondence to francois-xavier.ouf@irsn.fr

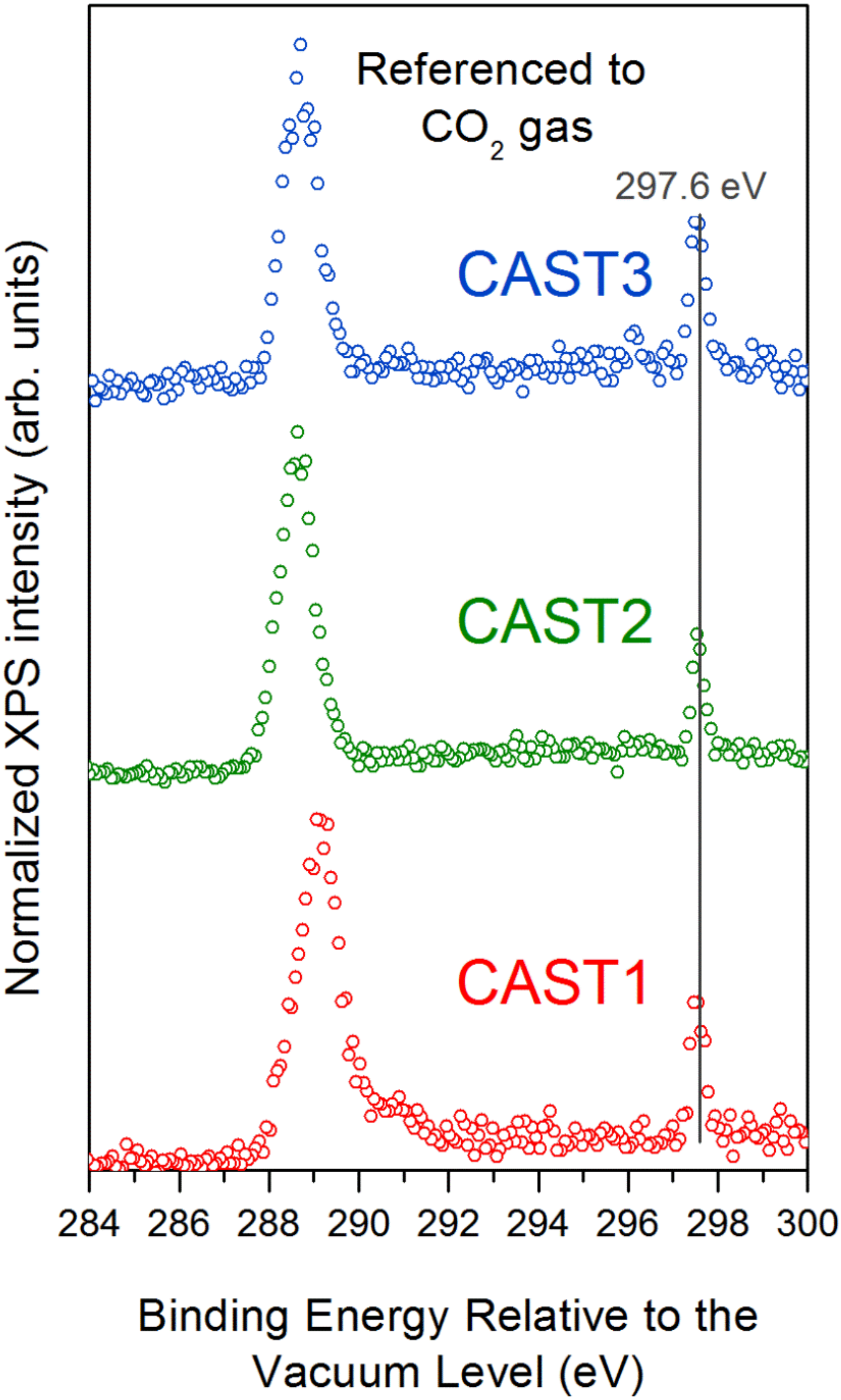

Figure S1: Comparison of C1s XPS spectra recorded on the aerosol phase at the PLEIADES beamline at SOLEIL for each set point and expressed in binding energy at the Fermi level (not corrected by work function).
